# Synergistic targeting of immunologic pathways to empower durable tolerance therapies

**DOI:** 10.3389/fimmu.2022.962177

**Published:** 2022-09-02

**Authors:** Gerald T. Nepom

**Affiliations:** Immune Tolerance Network and Benaroya Research Institute, Seattle, WA, United States

**Keywords:** immune tolerance, autoimmunity, regulation, clinical trials, immunotherapy

## Introduction

Therapeutic intervention to reverse immune-mediated disease faces several biological barriers, which correspond to stages of disease initiation and progression. Underlying disease susceptibility, whether due to genetic, maternal-fetal, or environmental causes, establishes highly variable platforms that differ among individuals and can underlie differential disease course and responses to particular therapies. The type of disease triggering event, whether infectious, allergen, or autoantigen, is also highly variable and establishes a pathogenic program at the tissue-immune interface that enlists multiple arms of immune response. If not self-limited, serious immune-mediated disease occurs when the evolution of these initiating factors develop into a progressive immunological disease involving a host of inflammatory and effector pathways. Opportunities to intervene therapeutically differ at each of these stages, and restoring homeostasis becomes progressively more difficult as an individual patient moves through these stages due to recruitment of additional arms of immune response, determinant spreading (expansion of the antigen/autoantigenic targets), and tissue response to injury, in which inflammation hinders regulation.

**Figure 1:**
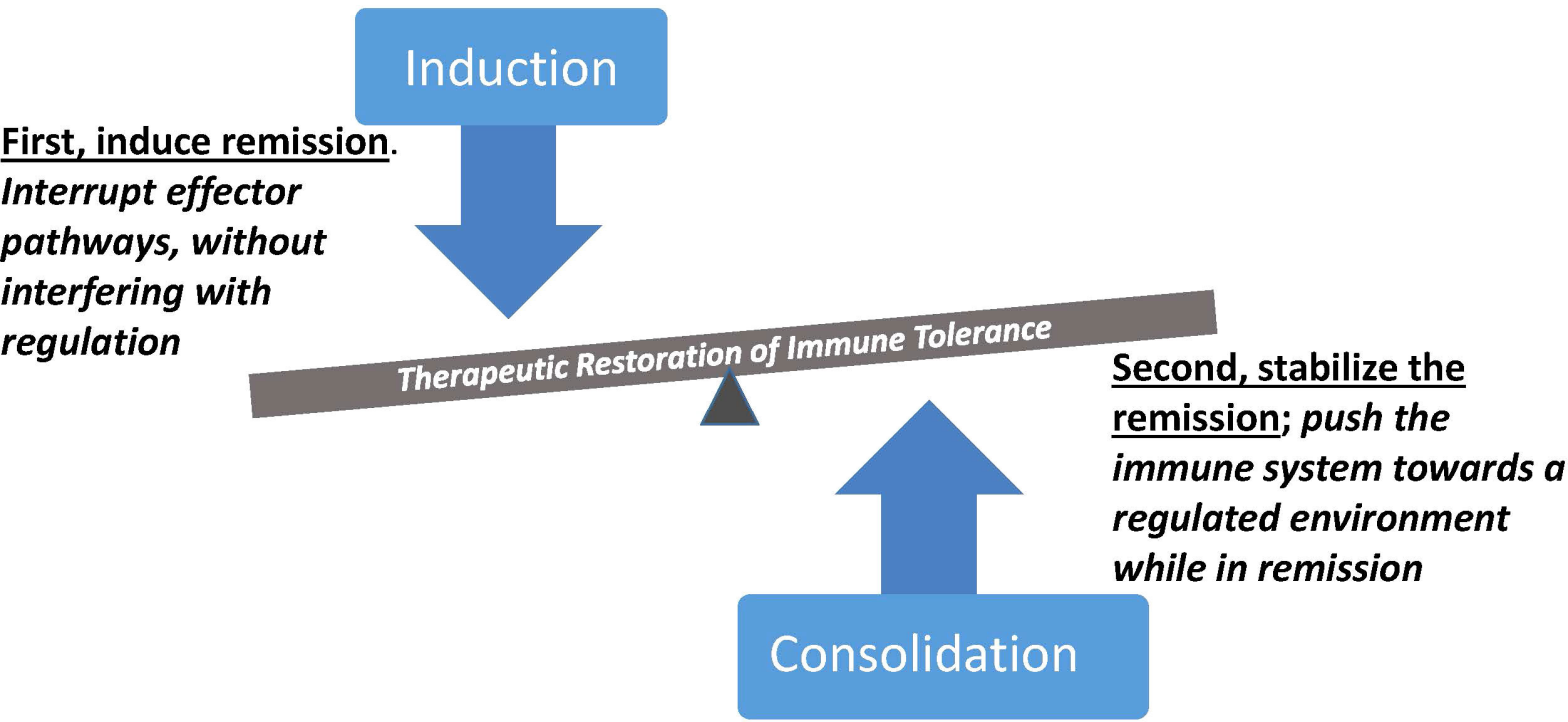
Synergistic contributions of immune therapies with complementary mechanistic targets. Induction of remission is initiated by abrogation of pathogenic effector cells and pathways, followed by consolidation therapy during remission to reinforce regulatory immune control. Selective therapeutic targeting is an important element of both steps, to avoid interfering with regulation during the inductive therapy and to avoid boosting pathogenic effector mechanisms during the pro-regulatory phase of treatment. Therapeutic outcomes with an immune regulatory dominance represent a form of immune tolerance with potential for sustained clinical benefit.

Over the last three decades, a diverse repertoire of immune therapeutics have been deployed, many capable of targeting selective elements within the immune system. At the same time, methods for monitoring components of the immune response have been introduced, and when used in the context of clinical trials they provide an opportunity to dissect pathways and types of responses to therapy that clarify the challenges posed when attempting to reestablish immune tolerance. The road to durable therapies, while still obscure overall, is being mapped through experience indicating that interrupting immune effector components with targeted intervention is often achievable but insufficient. Disease relapse mediated by persistent or recurrent effector mechanisms needs to be restrained, by combining remission-inducing therapy with therapy that supports regulatory immune mechanisms. Important implications that derive from this concept include selection of induction agents that will not impair desirable regulatory elements, and timing of regulatory therapies to take advantage of windows of remission when the tissue environment is conducive to reprogramming (**Figure 1**). This combination of modifying the autoreactive effector pathways while restoring homeostatic regulatory components is a mechanistically-oriented definition of immune tolerance. A more practical, clinically-oriented definition of tolerance useful for medical management is to achieve a state in which disease progression is stopped without requiring the use of general immunosuppression. In the examples which follow, clinical trials used this clinically-oriented definition as a measure of success, and parallel laboratory studies evaluated whether the mechanistically-oriented definition actually reflected the same outcomes.

### What does tolerance look like?

T lymphocytes drive many arms of the immune response, and are therefore often targets for depletional therapies in autoimmune and immune-mediated disease. Comparison of three drugs studied in very similar clinical trials of type 1 diabetes, the START, AbATE, and T1DAL trials, identifies key features that inform therapeutic mechanisms. In START [the Study of Thymoglobulin to Arrest Newly Diagnosed Type 1 Diabetes (1)], subjects received anti-thymocyte globulin to deplete cellular immune effector compartments as an attempt to arrest immune-mediated damage to target tissue and reestablish a naïve host immune environment. T cell depletion in this study was highly effective, with various peripheral effector cell populations showing diminished cell numbers after drug infusion. However, clinical and metabolic outcomes showed no benefit from drug administration, and a *post-hoc* analysis of immune biomarkers documented drastic loss of regulatory T cells as a bystander casualty of the potent T cell depleting therapy. In AbATE [Autoimmunity-Blocking Antibody for Tolerance (2)], a similar set of subjects received teplizumab, an anti-CD3 monoclonal antibody, as a more selective T cell modulating therapy. In contrast to START, in AbATE there was an overall average clinical and metabolic benefit in the treatment arm of the trial, although only 15% of those subjects showed durable benefit longer than 2 years. Analysis of the immune biomarkers in AbATE documented a novel mechanism, in which CD8 T cells characteristically showed upregulation of molecular and transcriptional indicators of cell exhaustion including PD1/TIGIT/KLRG1 and EOMES (3). In other words, instead of a depletional mechanism, teplizumab induced an agonist response that drove a developmental program towards a terminal nonpathogenic phenotype in most subjects in the trial. Notably, when these participants in the AbATE trial were re-treated with teplizumab one year after initial therapy, only the subjects with clinically beneficial responses showed a recurrent agonist exhaustion profile. This pathway was subsequently further elucidated in T1DAL [Inducing Remission in Type 1 Diabetes with Alefacept (4)], in which a comparable group of T1D subjects received alefacept, a LFA3-Ig fusion protein targeting CD2. In T1DAL, 30% of treated subjects achieved a durable benefit lasting 2 years, and mechanistic studies identified a dual mode of action that correlated with response. Alefacept treatment led to a moderate loss of CD2-positive lymphocytes from the T and NK compartments, depleting cells expressing high levels of surface CD2. However, T cells with low levels of CD2 showed an agonist response to drug, in which CD4 T cells acquired PD1 markers and were hyporesponsive to activation, while subsequent maturing CD8 T cells acquired two states characterized by exhaustion and regulatory markers, one set with TIGIT/PD1/EOMES and one set with CD57/KIR induction (5). Following discontinuation of alefacept therapy, both of these acquired CD8 properties correlated with beneficial clinical outcome. Notably, although teplizumab and alefacept drugs were initially developed because of presumed depletional activity directed against effector T cells, it is now apparent that peripheral depletion was incomplete, and the degree of depletion did not correlate with clinical outcome; instead, it was the partial agonist activity associated with exhaustion that correlated with response. Interestingly, in the alefacept trial, lymphocytes with high CD2 expression were indeed deleted, but the agonist activity on cells with lower CD2 expression appeared to be key; n addition, depletion of tissue-resident and tissue-associated effector T cells in these trials is unknown, but was likely not achieved. Two conclusions from these comparisons between related trials seem evident: First, T cell depletional therapy is not desirable if it does not allow regulatory cells to persist, and second, that modulation of effector cells accompanied by induction and persistence of a nonpathogenic regulated phenotype, rather than simply targeting for depletion, had the best outcome; in this respect, levels of surface expression of the target molecule (e.g., CD2) may be an important determinant of cell fate.

### Remission induction from immune deletion is not sufficiently durable

Several ITN trials in other autoimmune diseases reinforce the notion that immune therapies that solely interfere with pathogenic effector pathways are insufficient for durable benefit. In the successful trial of rituximab (anti-CD20) in ANCA-associated Vasculitis [the RAVE study (6)] that led to a new standard-of-care for this disease, many clinical relapses eventually manifest over time, suggesting incomplete effector deletion or inadequate regulation. Two subsequent trials in lupus nephritis were then designed, one combining induction using rituximab and cyclophosphamide with belimumab [anti-BAFF (7)] and another combining abatacept (CTLA4-Ig, targeting CD80/86) and cyclophosphamide [ACCESS (8)], to test alternative pathways to suppress recurrence of autoreactive B cells after depletional induction therapy. However, neither trial met their endpoints for clinical benefit, in spite of profound peripheral B cell depletion and, when belimumab was used, long-term persistence of B cell lymphopenia. In addition to raising doubts about heavily targeting the B cell compartment, biomarker studies in ACCESS implicated abatacept in a potentially undesirable outcome, namely a decrease in regulatory T cells. Specifically, in addition to reducing numbers of circulating activated follicular T helper cells (a desirable and expected outcome), costimulatory blockade with abatacept also substantially depleted the pool of peripheral CD4 regulatory T cells, particularly those with memory cell characteristics. This apparent interference with regulatory T cell pathways was recapitulated in the ACCLAIM trial [A Cooperative Clinical Study of Abatacept in Multiple Sclerosis (9)], in which abatacept therapy failed to demonstrate clinical efficacy while at the same time reducing numbers of both circulating follicular T cell helper and regulatory cells.

The PAUSE trial [Efficacy of Ustekinumab followed by Abatacept for the Treatment of Psoriasis Vulgaris (10)] was another attempt to sustain clinical remission by targeting costimulation pathways. In this study, psoriatic patients with initial successful induction therapy using ustekinumab (anti-IL12/23 p40) were then randomized to receive either abatacept or another course of ustekinumab. Abatacept again failed to prolong remission, and transcriptional signatures of epidermal inflammatory pathways were not suppressed by abatacept, correlating with clinical relapse. In other words, not only did abatacept interfere with regulatory T cell potential, but key target cell inflammation cascades were not disrupted.

Lessons learned from these ‘negative’ clinical trials reinforce the concept that immunosuppression that interferes with regulation has undesirable consequences for immune tolerance, even in the context of purposeful effector cell targeting. Together with the favorable function of teplizumab and alefacept as agonists reprogramming T cells towards hyporesponsive states and sparing regulatory cells, these studies point us towards needing strategies that will enhance regulatory pathways and sustain inhibition of effector pathways for more durable outcomes.

### Selectively boosting regulation following induction of remission

Compared to the number of potential therapies that target effector cell pathways, the development of pro-regulatory therapeutics is quite limited, but is an area primed for rapid expansion. One attractive drug target is the high affinity IL2 receptor expressed on regulatory T cells. Several IL2 agonist formulations are in clinical development that are designed for preferential targeting of this pathway, with various design strategies to avoid activation of effector T and NK cells that are potentially pathogenic (11). The need for this type of selective targeting was emphasized by the results of an initial clinical trial using IL2 as a therapeutic modulator in recently-diagnosed T1D (12). In this trial, expansion of regulatory T cells was observed in all treated subjects over a 30 day course of IL2 therapy combined with rapamycin, and sustained phosphorylation of STAT5, part of the signaling cascade induced by IL2, was documented in the regulatory T cells in peripheral blood samples. However, the trial was terminated early due to unexpected rapid loss of c-peptide during the 30 day treatment period, an adverse outcome that implied potential acceleration of disease. Biomarker studies of these subjects documented increases in NK effector populations during this same treatment interval, an outcome most likely corresponding to expansion of CD25 (IL2R) –positive NK cells, rather than an effect of the rapamycin, therefore presumed to indicate a likely IL2 stimulatory effect, negating the intended targeting of the IL2 receptor on regulatory cells.

This study indicated that the balance between supporting regulatory cell function while at the same time not enhancing inflammatory effector cell activation is a precarious challenge, with important implications for clinical therapy. While creative drug design should be helpful in improving selectivity for boosting regulatory responses, a direct approach of combining regulatory therapy with complementary anti-effector therapy is a practicable strategy, particularly for disease indications in which remitting therapies already exist or are under development. In this context, envisioning IL2 as a pro-regulatory drug illustrates the important requirement for selectivity of mechanisms of action, with a need to monitor and avoid expansion or activation of pathogenic pathways.

There are also a number of alternative strategies to achieve a therapeutic consolidation effect, directed at expanding regulatory cells or using tactical exposure to antigens for regulatory enrichment. For example, adoptive cell therapies using Tregs that are expanded using *ex vivo* antigen stimulation may be feasible for diseases where immunodominant antigens are known, and creative molecular engineering of these cells to encourage function and stability is conceptually feasible (13, 14). Similarly, transfer of antigen-specific TCR into committed Tregs, or design of chimeric antigen receptor (CAR) Tregs, perhaps engineered with both antigen specificity and a regulatory phenotype, may be tools to increase durability of remission (15, 16). Distinct from these cell therapies, use of autoantigen delivery systems that elicit regulatory responses are currently being explored, with some early clinical trials underway. Various non-immunogenic delivery systems and potential tolerogenic adjuvants, including tissue-targeting fusion proteins, nanoparticles (17) and red cell carriers of antigen (18), are under active exploration and development for clinical use (19).

It will be important to learn whether these ways of delivering antigen are capable of directing a regulatory response in patients with active autoimmune disease, and whether they will be generally safe and efficacious used as a consolidation strategy following initial induction of remission when the tissue environment is more receptive to regulatory modulation. In preclinical studies, mechanisms attributed to antigen-delivery therapies include deletional effects on effector populations, trafficking modification of pathogenic tissue entry, induction of T cell exhaustion or anergy, modulation of antigen presentation, and enhancement of regulatory T cell numbers (18, 20–23). Clinical trials with autoantigen delivery have generally demonstrated safety and some immunomodulatory effects (recently reviewed elsewhere, e.g (24, 25).,), but emergence of homeostatic regulatory mechanisms responding to tissue-specific and disease-relevant antigens will be critical for durable efficacy. A recent clinical example, albeit in a small number of subjects, was the combination of rituximab (anti-CD20) with autologous regulatory T cells, which showed partial preservation of c-peptide in a study in adolescents with type 1 diabetes (26). Strategies that first induce clinical remission should foster a tissue environment more conducive to regulatory responses, positioning antigen-delivery therapeutics as a potential partner in a therapeutic combination with synergistic mechanisms, for example when used in combination with an induction agent that removes pathogenic effector cells or that provides active immunomodulatory signals that support tolerogenic antigen recognition.

## Discussion

As outlined in a recent review of multiple studies from the Immune Tolerance Network (27), using a mechanistic approach to classify therapeutic intervention in immune-mediated diseases into (i) interrupting effector mechanisms, (ii) restraining innate activation, or (iii) boosting regulation, presents an opportunity to address challenges posed by persistent immunologic memory and deficient immunologic regulation in diverse clinical contexts. It is now timely to translate these mechanism-focused observations into practicable therapeutic strategy. Achieving durable immunological perturbation induced by targeted therapy and aligning these changes with clinical remission in the absence of general immunosuppression is informing and defining a future for successful immune tolerance. By analogy with two-step therapies that are commonly used in oncology indications, it is timely to envision initiating therapy in autoimmunity by using agents that induce remission, followed by consolidation therapies that extend the durability of this remission. Initial remitting drugs may target pathogenic effector pathways *via* depletional, deviation, anergy or exhaustion mechanisms, but as illustrated in studies cited above, these inductive therapies need to avoid interfering with immune regulatory elements to enable tolerance outcomes. The subsequent consolidation strategy, designed to stabilize the initial remission, must be complementary to this strategy and be supportive of regulatory components while avoiding activation of pathogenic pathways. Treating patients while in disease remission with novel pro-regulatory therapies will require a new way of clinical thinking for most immune-mediated diseases, contrasting with current medical practice patterns of waiting for relapse before additional treatment. Nevertheless, a relatively quiescent tissue environment may be necessary in order to favor development of robust regulatory immune states capable of suppressing disease recurrence. While conceptually the induction and consolidation steps in this therapeutic model mechanistically are sequential, it is also possible that combination therapy with agents addressing both of these steps could be used simultaneously. This scenario is consistent with the mechanistic studies in the teplizumab and alefacept studies that showed potential for single therapeutic agents to inhibit effector pathways while also acting as agonists for regulatory elements of the immune response. Clinical trials combined with mechanistic studies that explore enhancing durability of remission through synergy with selective regulatory therapy offer the potential for restoration of immune homeostasis, a challenging but achievable goal.

## Author contributions

The author confirms being the sole contributor of this work and has approved it for publication.

## Funding

The Immune Tolerance Network studies described in this article were supported by the National Institute of Allergy and Infectious Diseases of the National Institutes of Health under Award Number UM1AI109565. The content is solely the responsibility of the author and does not necessarily represent the official views of the National Institutes of Health.

## Acknowledgments

Many colleagues at Benaroya Research Institute and in the Immune Tolerance Network (ITN) have contributed data and ideas that have been critically important in the evolution of the concepts discussed in this article. Additional descriptions can be found at https://www.immunetolerance.org/researchers/strategic-plans, which presents a summary of therapeutic strategies developed as a collaborative effort by the Network Steering Committee of the ITN.

## Conflict of interest

The author declares that the research was conducted in the absence of any commercial or financial relationships that could be construed as a potential conflict of interest.

## Publisher’s note

All claims expressed in this article are solely those of the authors and do not necessarily represent those of their affiliated organizations, or those of the publisher, the editors and the reviewers. Any product that may be evaluated in this article, or claim that may be made by its manufacturer, is not guaranteed or endorsed by the publisher.
